# Leaf age structures phyllosphere microbial communities in the field and greenhouse

**DOI:** 10.3389/fmicb.2024.1429166

**Published:** 2024-08-14

**Authors:** Julie K. Geyer, Rita L. Grunberg, Jeremy Wang, Charles E. Mitchell

**Affiliations:** ^1^Department of Biology, University of North Carolina at Chapel Hill, Chapel Hill, NC, United States; ^2^Department of Genetics, University of North Carolina at Chapel Hill, Chapel Hill, NC, United States; ^3^Wilson Center for Science and Justice at Duke Law, Durham, NC, United States; ^4^Environment, Ecology, and Energy Program, University of North Carolina at Chapel Hill, Chapel Hill, NC, United States

**Keywords:** succession, microbiome, phyllosphere, metabarcoding, mycobiome

## Abstract

The structure of the leaf microbiome can alter host fitness and change in response to abiotic and biotic factors, like seasonality, climate, and leaf age. However, relatively few studies consider the influence of host age on microbial communities at a time scale of a few days, a short time scale relevant to microbes. To understand how host age modulates changes in the fungal and bacterial leaf microbiome on a short time scale, we ran independent field and greenhouse-based studies and characterized phyllosphere communities using next-generation sequencing approaches. Our field study characterized changes in the fungal and bacterial phyllosphere by examining leaves of different relative ages across individuals, whereas the greenhouse study examined changes in the fungal microbiome by absolute leaf age across individuals. Together, these results indicate that fungal communities are susceptible to change as a leaf ages as evidenced by shifts in the diversity of fungal taxa both in the field and the greenhouse. Similarly, there were increases in the diversity of fungal taxa by leaf age in the greenhouse. In bacterial communities in the field, we observed changes in the diversity, composition, and relative abundance of common taxa. These findings build upon previous literature characterizing host-associated communities at longer time scales and provide a foundation for targeted work examining how specific microbial taxa might interact with each other, such as fine-scale interactions between pathogenic and non-pathogenic species.

## Introduction

Plants live in association with microbial communities, including bacteria and fungi, which influence plant fitness by modulating disease resistance, drought tolerance, and nutrient uptake (Balint-Kurti et al., 2010; Hubbard et al., 2014; Schlaeppi and Bulgarelli, 2015; Busby et al., 2016) – these microbes inhabiting plant leaves are collectively known as the “phyllosphere microbiome”. The structure of the phyllosphere microbiome has the capacity to respond to drivers across a broad range of temporal scales, and there have been many studies examining how host age shapes plant leaf and root bacterial communities (Lerner et al., 2006; Xu et al., 2009; İnceoğlu et al., 2011; Chaparro et al., 2013; Wagner et al., 2016; Biget et al., 2021; Doherty et al., 2021). The literature on the phyllosphere microbiome is imbalanced; research on how age structures plant fungal communities is less common and less consistent – one study showed that fungi in the rhizosphere are shaped by host plant age (Biget et al., 2021), and another demonstrated that age has no effect on the diversity of fungi in plant leaves and roots (Doherty et al., 2021). In addition, leaf age can drive the prevalence and severity of infection by fungal pathogens (Halliday et al., 2017), so leaf or plant age may be important determinants of interactions between pathogens and non-pathogens (Bruns et al., 2022).

There are several important considerations in studies examining changes in the plant microbiome as a host ages, like the temporal scale of the study. The appropriate temporal scale to understand the dynamics of an ecological community depends on how quickly the organisms can respond to variation in conditions – for microbes, that scale can be on the order of minutes, hours, and days. However, relatively few microbiome studies explore this fine-scale variation (but see Maignien et al., 2014). It remains important to explore changes in the diversity, community structure, and relative abundance of microbial species at the community level, which would provide a foundation for asking and answering targeted questions about the ecological dynamics of microbial ecosystems.

Likewise, exogenous variables, like climate variation and seasonality may confound studies examining the effects of age on microbiota (Meyer and Leveau, 2012). Climate varies over time and as a plant ages, which can confound the effects of host age on microbial communities. To accurately examine the influence of host age, it is important to separate the endogenous effects of age from other sources of temporal variation, which can be achieved through controlled greenhouse studies. Temporal scale refers to the time frame over which ecological and biological processes occur, and many studies examining variation in the plant microbiome as a host ages consider the importance of temporal scale (Maignien et al., 2014; Wagner et al., 2016; Doherty et al., 2021). However, variation in microbial communities can occur within minutes or days, and this temporal scale is not often reflected in the literature on the plant microbiome.

Conceptual frameworks from community ecology posit that four fundamental processes drive variation in community structure: dispersal, ecological selection, drift, and speciation (Vellend, 2016). Species richness, which refers to the number of unique species in a given area, is determined by a balance of colonization (dispersal) and extinction (drift), and the size of a host individual predicts the number of microbial species it can support (MacArthur and Wilson, 1967). As a leaf ages, several physiological changes can occur, like increased leaf length and thickness, increased cuticle thickness, and decreased nutrient content (Mauseth, 2012). Furthermore, older leaves have been exposed to their environment for a longer period, providing more opportunities for microbial colonization, which is expected to increase microbial richness (Maignien et al., 2014; Christian et al., 2015). Many of the potential changes in microbial communities that may occur alongside the physiological changes associated with leaf maturation remain unclear.

To fill these gaps in the literature, and in particular, explore fine-scale variation in the plant microbiome, we ran separate, complementary field and greenhouse-based studies to examine how changes in the leaf microbiome might relate to host age. The field study, which was limited to two sampling days allowed us to examine microbial communities shaped by both host (endogenous) and environmental (exogenous) factors. Additionally, our field study allowed us to analyze both fungal and bacterial communities – a dataset that allows for a more comprehensive picture of how diverse micro-communities may change as a host ages. The greenhouse experiment, which was limited to fungi, allowed us to explore how host age shapes microbial communities in absence of the additional layer of environmental variation. The greenhouse study also allowed us to sample leaf fungal communities on time scales that may more accurately reflect the biology of microorganisms.

## Materials and methods

Leaves were collected in a greenhouse environment as well as a field environment to test whether leaf age structures microbial communities. The field environment represented an opportunity to examine natural microbial communities potentially shaped by environmental conditions, whereas the greenhouse environment represented an opportunity to examine changes in the host microbiome, largely in absence of climate variation and at short time scales. The specific field location was chosen because of the abundance of tall fescue (*Lolium arundinaceum*) and because it has been well-characterized both in terms of foliar fungal parasites and host plant community composition (Halliday et al., 2017, 2018; O’Keeffe et al., 2021; Grunberg et al., 2023).

### Experiment 1: field leaf collections

**FIGURE 1:**
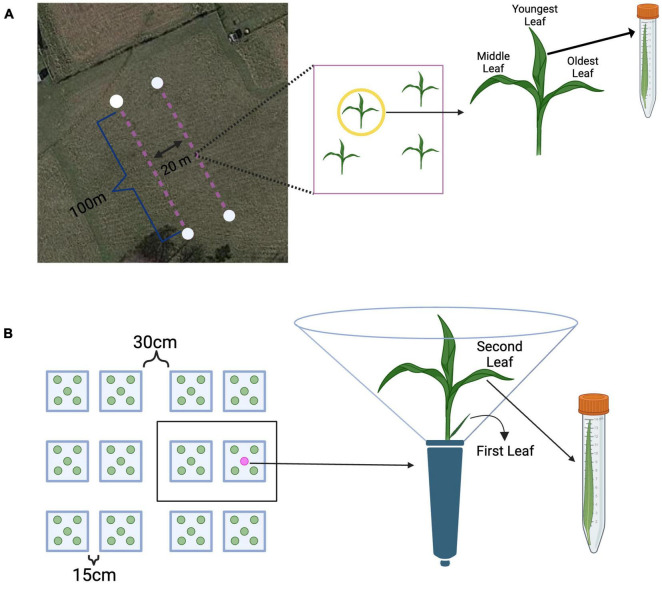
Methods overview from the field and greenhouse. **(A)** Leaves were sampled from plants along two 100 m transects. Each transect had 20 possible sampling points. Each leaf from a tiller was collected in a pre-determined random order, placed into a sterile centrifuge tube, and immediately placed on ice. Source: Map data © 2022 Google Earth/Airbus, reproduced according to Geo Guidelines. **(B)** All plants were grouped into 31 spatial blocks, each having 10 plants (represented as a black box). On each sampling day, the second leaf was collected was collected from approximately 20 randomly selected plants. Each leaf was measured for length, clipped into an individual sterile centrifuge tube, and immediately placed on ice. Created with BioRender.com.

Field samples were collected on October 29^th^ and 30^th^ of 2019 at Widener Farm in the Duke Forest Teaching and Research Laboratory (Orange County, NC, USA) along two 100m transects 20m apart to ensure independence and minimize spatial autocorrelation – a total area of roughly 400m^2^ was surveyed. Each sampling point was at least 10m apart to ensure independence. Leaves from the first transect were collected from 17 unique sampling points (sampled on October 29^th^), and the second transect (sampled on October 29^th^ and 30^th^) had 13 sampling points. Leaves were collected from each tiller based on their relative age, which can be determined visually; the oldest leaves are the outermost leaves growing from the base of the plant, and the youngest leaves are the innermost leaves growing from the base of the plant (Figure 1A). Average monthly temperature at Duke Forest in October of 2019 was roughly 18°C, average humidity was 77%, and average precipitation was roughly 9.5cm (Western Regional Climate Center, 2021). The temperature during sampling on October 29^th^ ranged from roughly 16–22°C and weather conditions were overcast. On October 30^th^, the weather was overcast and the approximate temperature during sampling was 22°C. At each sampling point, a random tiller was selected for sampling, and all leaves were collected (in a pre-determined random order) and stored in individual sterile centrifuge tubes, immediately put on ice (Figure 1A). To standardize tall fescue tillers’ developmental stage and infection treatment, we sampled tillers with three leaves and without lesions (indicative of active pathogen infection). In total we sampled 96 leaves from 32 tillers, of which 84 were randomly selected for sequencing. All leaves were returned to the lab, measured for leaf length, and stored in a −80°C freezer until DNA extractions.

### Experiment 2: greenhouse set-up and leaf collections

*Epichloë*-free tall fescue seed of a single cultivar, KY-31, (obtained from Dr. Tim Phillips, University of Kentucky) was used in this experiment to avoid inter-individual variation in *Epichloë* infection – transmission through seeds is less than 100% and testing infection status requires harvesting a whole tiller, which could not have been done until the end of our experiment. Tall fescue is commonly asymptomatically infected with *Epichloë coenophiala*, one of many systemic, vertically transmitted, fungal endophytes which form symbioses in the above-ground host tissues of temperate grasses (White et al., 2021) – endophyte-free seed lacks this symbiont and is widely used in place of endophyte-infected seed to reduce livestock toxicity (Young et al., 2014). To boost the germination rate, seeds were first primed by soaking all seeds in sterile water for 4 h, followed by drying overnight in a sterile biosafety cabinet. This seed was then surface sterilized following priming by agitating seed in a solution of 20% bleach and 1% tween20 for 12 min. Seed was then washed thoroughly with sterile water seeds were transplanted into autoclaved Sungro Metromix 360 soil in 5.08 × 17.78 cm (2 × 7”) plastic conetainers (Stuewe and Sons Deepots, Corvallis, OR, USA) with 5 beads of Osmocote (to provide continuous, slow release of nitrogen, phosphate, and potassium). Each conetainer received only 1 seed, and there were a total of 800 plants in individual conetainers.

To prime host individuals in the greenhouse with microbes from their natural environment and create a “microbial slurry”, field soil was collected on November 3^rd^, 2019, from no more than 5 cm below tall fescue in the field. 225g field soil was immediately returned to the lab where it was soaked in 1.5 liters autoclaved diH_2_O and agitated every 5 min for a total of 30 min. This solution was then vacuum filtered through a 2.5 μm Grade 5 Whatman qualitative filter paper into an autoclaved flask. Filtration grade was chosen to exclude soil-borne fungal pathogens (Zabel and Morrell, 2020). This single microbial slurry was homogenized and applied to all experimental plants on the same day it was prepared.

To maintain growth of the plants, each conetainer was watered to saturation three times per week and plants were kept under full spectrum high-pressure, sodium lights (from 9:00am to 7:00pm, as determined by ambient light availability) in addition to receiving natural light. Growth conditions in the greenhouse were set to 70.0°F (21°C), and full-spectrum lights were triggered to switch on when the light in the greenhouse dropped below 350 W/m^2^ and turn off when natural light rose above 600 W/m^2^. Plants were monitored daily until the second leaf emerged, roughly 1 month after seeds were primed and planted – at which time the leaves were marked at the base with a leaf tag. To standardize leaf age in addition to plant age, only plants where the second leaf emerged on the same day were used. All 490 plants not meeting this criterion were discarded prior to sampling. All remaining 310 plants were grouped into 31 spatial blocks, each block having 10 plants – approximately 20 plants were randomly sampled (harvested) across all spatial blocks on each sampling day. Upon transplantation, each conetainer was inoculated with 2mL of the microbial slurry (detailed above) while avoiding contact with the plant. Due to the high volume of host individuals in the greenhouse, additional measures were taken to ensure independence – each plant conetainer received a clear acetate divider around the conetainer to ensure that plants did not touch as they grew, while allowing sufficient light to reach the plant. The second leaf was chosen for sampling as it represents the next youngest leaf on a tiller that has the capacity to mature and grow – the first leaf on a tiller does not grow as long and has a notably shorter lifespan (Figure 1B).

Sampling of the remaining 310 plants began after the second leaf emerged on November 20th, roughly 1 month after seed was primed and planted. Sampling occurred three times per week (Monday, Wednesday, and Friday) and on each sampling day, approximately 20 plants were randomly selected (without respect to block) and from each of those plants, the second leaf was collected. Sampling times were chosen because they represent a temporal scale on which microbial communities can vary, but has not yet been explored in the literature. Each leaf was measured for length, clipped into an individual sterile centrifuge tube, measured, and then stored at −80°C until DNA extractions. After a leaf was collected, the sampled plant was left in place and marked so that it would not be re-sampled – this was to ensure that the surrounding plant community remained constant throughout the duration of the experiment. By the end of the experiment, 295 of the 310 plants had been randomly selected and sampled, leaving 15 plants that were not sampled – these extra plants were included in the experiment from the beginning to ensure there would be enough individuals to sample if a portion of host plants died during the month of sampling.

### DNA extraction and sequencing

DNA extraction was performed on samples from the field and the greenhouse to prepare them for library preparation and sequencing. Samples were taken from a −80°C freezer and immediately placed in liquid nitrogen for 1 min, followed by manual grinding with sterile pestles. Of the 96 field samples that were collected, 84 were randomly selected (across all age groups) for DNA extraction and sequencing. Of the 295 greenhouse samples that were collected, 102 samples were randomly selected (across all age groups) for DNA extraction and sequencing. Once each leaf was ground into a fine powder, the sample was put back in a −80°C freezer. DNA from both sampling efforts was extracted using the DNEasy PowerSoil kit following manufacturer (Qiagen) instructions. Extracted samples were frozen at −80°C until library preparation for sequencing.

To prepare fungal libraries from extracted DNA, the Zymo Quick-16S NGS Library Prep Kit was customized with ITS1-F and ITS2 fungal primers (White et al., 1990; Smith and Peay, 2014). The Internal Transcribed Spacer (hereafter, ITS) region is a highly conserved region of spacer DNA and is commonly used for identifying fungal species. ITS1-F and ITS2 primers were selected according to the Earth Microbiome Project’s ITS Illumina Amplicon Protocol. Following manufacturer instructions, this kit was used to amplify fungal DNA, attach index primers, quantify nucleic acids, and pool samples. A spike in of 15% phiX was used to increase sample heterogeneity and improve sequencing output. The High Throughput Sequencing Facility at UNC performed all sequencing using the Illumina MiSeq platform.

To prepare bacterial 16S libraries from extracted DNA, samples were amplified using 27F and 1492R 16S primers (Frank et al., 2008) with 1:100 w/w genomic DNA of *Salinibacter ruber* as a spike-in control. PCR blockers (PNA Bio Inc.) were used to block the amplification of host mitochondrial and chloroplast DNA resulting in a final concentration of 1 μM mPNA and pPNA per sample well. PCR amplicons were barcoded and prepared for multiplex sequencing using the Oxford Nanopore Native Barcoding Kit (SQK-NBD112.6). Libraries were sequenced using two R10.4 flow cells on MinION Mk1B.

### Bacterial and fungal community analysis

Bacterial nanopore reads were basecalled, demultiplexed, and trimmed using Guppy v6.3.8. Minimap2 (Li, 2018) was used to align trimmed reads to rrnDB v5.8 (Stoddard et al., 2015) and alignments were processed using custom code to construct taxonomic abundance profiles. All raw fungal sequence reads were demultiplexed using the Illumina bcl2fastq pipeline (v.2.20.0) and sequence adapters were removed in QIIME2 using Cutadapt (version 2.9). Fungal amplicon sequence variants (hereafter, ASVs) were assigned to sequencing output using DADA2 (Callahan et al., 2016) and sequences below quality score 15 were removed. Taxa were matched to the UNITE fungal ITS database (version 8.99, released on April 2^nd^, 2020) in QIIME2 (Kõljalg et al., 2013). Sequencing reads generated from the Illumina MiSeq platform were classified as ASVs to provide higher resolution taxonomic data. Nanopore-generated reads were classified as operational taxonomic units (hereafter, OTUs) due to the higher error rate of nanopore sequencing. For both 16S and ITS reads, all samples with fewer than 1,000 reads were filtered out. Then, all samples were rarefied using the QIIME2 plugin ‘alpha-rarefaction’ to a uniform sampling depth that was chosen for each dataset to approximate the remaining samples’ lowest read count. For each dataset, rarefaction was performed at multiple thresholds to confirm consistent results. Specifically, the ITS greenhouse and 16S field datasets included samples with read counts down to around 1,000 while the ITS field samples consistently had read counts over 6,000. Thus, data were rarefied to 1,000 reads/sample for ITS greenhouse data, 1,000 reads/sample for 16S field data, and 6,000 reads/sample for ITS field data.

### Statistical analyses

To examine patterns of microbial diversity by leaf age in the field, community metrics, like richness and Shannon diversity were calculated in QIIME2 for both 16S and ITS reads, leaving us with 84 samples (out of 84 original samples) that were analyzed for bacterial Shannon diversity, richness, and Bray-Curtis dissimilarity, and 76 samples (out of 84 original samples) that were analyzed for fungal Shannon diversity, richness, and Bray-Curtis dissimilarity. Both richness and Shannon diversity were used to assess changes in diversity as Shannon diversity accounts for the relative abundance of taxa in addition to the number of unique taxa present. For diversity analyses, as well as community composition analyses, transect and timepoint were combined into a single variable with 3 levels to account for the fact that timepoint sampled was confounded with transect sampled – these 3 levels were: sampling timepoint #1_transect #1, sampling timepoint #1_transect #2, and sampling timepoint #2_transect #2. Leaf age was coded as a continuous variable in the greenhouse dataset. Separate models for bacterial and fungal data were run to understand the relative importance of age in structuring these two domains. To account for repeated sampling of individual tillers, as well as the combined effect of transect and timepoint sampled, we ran mixed effects models (using lmerTest version 3.1-3, function ‘lmer’). Our final models included leaf length and age as fixed effects and tiller ID, transect, timepoint, and sampling block as intercept random effects. Model fit was assessed via diagnostic plots and visual inspection of residuals. To understand how leaf age influenced the Shannon diversity of fungi and bacteria in the field, we used a model statement in the form:

Leaf Age × Leaf Length + *1| Transect/Timepoint (combined variable) + 1| Tiller ID*.

To understand how leaf age influenced the richness of fungi and bacteria we used model statements in the forms:

Leaf Age × Leaf Length + *1| Transect/Timepoint (combined variable) + 1| Tiller ID* and

Leaf Age + Leaf Length + *1| Transect/Timepoint (combined variable) + 1| Tiller ID*, respectively.

Lastly, we used a model statement in the form:

Leaf Age + Leaf Length + *1| Sampling Block*

to understand how age structured fungal Shannon diversity and fungal richness in the greenhouse. We also examined pairwise comparisons of least squares means (using emmeans version 1.8.4-1, function ‘ls_means’). η^2^_partial_ was calculated as a measure of effect size for leaf length and leaf age as response variables to fungal diversity and richness (using effectsize version 0.8.3, function ‘F_to_eta2’). Both pairwise comparisons, effect sizes, as well as the mixed effects models were computed in R.

To examine patterns of bacterial and fungal community structure by leaf age and timepoint, Bray-Curtis distance matrices were constructed for each community using QIIME2. Patterns in community dissimilarity were visualized using a principal coordinate analysis (PCoA) (McMurdie and Holmes, 2014) – each independent variable (leaf age and timepoint) was implemented in separate models for both fungi and bacteria. We used a permutational multivariate analysis of variance (PERMANOVA) on Bray-Curtis distances to test if differences in community structure were related to relative leaf age, sampling transect, and timepoint. Permutations were restricted within a tiller to account for resampling of tillers (n permutations = 999). The PERMANOVA was run in R using the ‘adonis2’ function in the vegan package 2.6–4. A betadisper test (using the ‘betadisper’ function in the vegan package 2.6–4) was also run to test for homogeneity of variances.

To examine changes in the relative abundance of bacterial and fungal genera by relative leaf age, taxonomy counts of fungal and bacterial genera (computed using QIIME2) were integrated in multivariate generalized linear models (function ‘manyglm’) with a negative binomial distribution. We ran separate models for fungal and bacterial communities. Our models were limited to include the 20 must abundant fungal and bacterial genera, while also filtering out unidentified taxa, to increase our ability to detect changes in specific genera (as per Mason et al., 2022). To account for repeated sampling within tillers, permutations were restricted within a tiller using the ‘bootID’ argument (*n* = 999 permutations) (Wang et al., 2012). Multivariate GLMs allowed us to detect genus-level (univariate) responses to differences in leaf age. Univariates tests were adjusted for multiple comparisons using a Holm step down procedure. GLMs were run using the mvabund package (version 4.2.1) in R (Wang et al., 2012).

## Results

Of the field samples surveyed for fungal diversity and community structure, Illumina sequencing of ITS generated 5,470,071 reads, of which 4,012,642 passed quality filtering. Each sample had an average of 47,769 reads per sample. After rarefaction, using DADA2, we identified 2,484 unique amplicon sequence variants, hereafter, ASVs (fungal taxa). Of the field samples surveyed for bacterial diversity and community structure by sequencing 16S, we generated roughly 20Gb data, an average of 131,969 reads passing basecall QC per sample from 2 MinION flow cells; after primer identification and trimming, an average 98,204 per sample remained for taxonomic analysis. After rarefaction, using MiniMap2, we identified 2,698 OTUs. Although not all reads could be identified down to the genus level, all ITS reads (from both the greenhouse and field) aligned to the kingdom fungi, and all 16S reads aligned to the kingdom bacteria. Most fungal reads belonging to field samples classified to the genera *Cladosporium, Hannaella, and Articulospora* and most bacterial reads from field samples classified to the genera *Rhizobium, Aurantimonas, and Pedobacter; Cladosporium spp.* also made up a notable portion of total fungal reads from samples in the field (see Supplementary Tables 1, 2).

### The influence of leaf age on fungal richness and diversity depended on leaf length in the field

**FIGURE 2:**
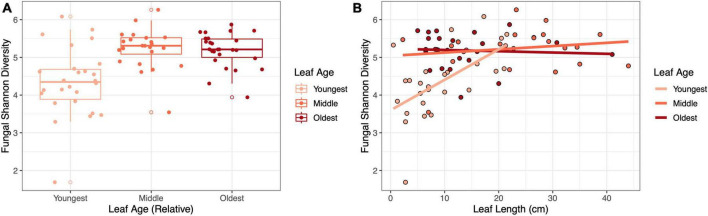
The influence of leaf age on fungal diversity depended on leaf length in the field. Panels show **(A)** the effect of relative leaf age on fungal Shannon diversity (*p* < 0.001) and **(B)** the effect of leaf length and leaf age on fungal Shannon diversity (*p* < 0.001).

Leaf age and length were important factors that interacted to shape fungal diversity in the field. In terms of Shannon diversity, the youngest leaves were the least diverse (LMM, age: *F*_2,61.9_ = 19.27, *p* < 0.001, η^2^_partial_ = 0.38; Figure 2A), and diversity increased with leaf length (LMM, leaf length: *F*_1,70_ = 9.70, *p* = 0.003, η^2^_partial_ = 0.12); moreover, youngest leaves that were longer approached the diversity of the older leaves (LMM, age × length: *F*_2,65.4_ = 8.71, *p* < 0.001, η^2^_partial_ = 0.21; Figure 2B). A similar, yet stronger, main effect of leaf age (LMM, age: *F*_2,59.1_ = 39.44, *p* < 0.001, η^2^_partial_ = 0.57), main effect of leaf length (LMM, *F*_1,69.89_ = 14.36, *p* < 0.001, η^2^_partial_ = 0.17) and interaction occurred for fungal richness (LMM, age × length: *F*_2,62.8_ = 15.35, *p* < 0.001, η^2^_partial_ = 0.33; Supplementary Figure 1).

### Leaf age influenced bacterial diversity, but not bacterial richness, in the field

**FIGURE 3:**
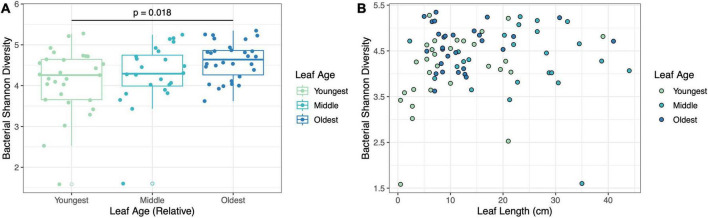
Leaf age influenced bacterial diversity but was not dependent on leaf length in the field. Panels show **(A)** the effect of relative leaf age on bacterial Shannon diversity (*p* = 0.03) and **(B)** the effect of leaf length and leaf age on bacterial Shannon diversity (*p* = 0.22).

Bacterial Shannon diversity was influenced by relative leaf age (LMM, *F*_2,64.7_ = 3.65, *p* = 0.03) (Figure 3A), but not leaf length (LMM, *F*_1,70.2_ = 2.67, *p* = 0.10) (Figure 3B) – older leaves had higher bacterial diversity. Likewise, there was no interaction between leaf length and relative leaf age (LMM, *F*_2,68_ = 1.55, *p* = 0.22) (Figure 3B). Pairwise comparisons indicated that the oldest leaves showed a tendency to be more diverse than the middle leaves (Tukey HSD; *p* = 0.07) and were significantly more diverse than the youngest leaves (Tukey HSD; *p* = 0.018) (Figure 3A). Bacterial richness was not influenced by either relative leaf age (LMM, *F*_2,77_ = 1.48, *p* = 0.23) or leaf length (LMM, *F*_1,77_ = 0.16, *p* = 0.69) (Supplementary Figure 2).

### Leaf age influenced fungal and bacterial community composition in the field

**FIGURE 4:**
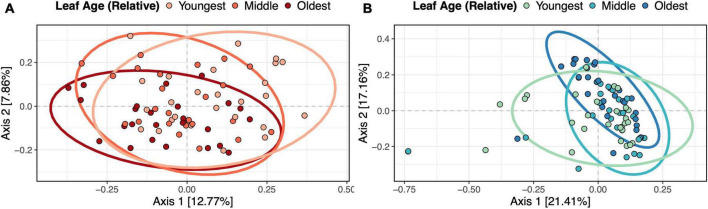
Relative leaf age influenced fungal and bacterial community composition in the field. Panels show **(A)** fungal and **(B)** bacterial Bray-Curtis dissimilarity with ellipsoids (95% confidence intervals) drawn for each relative leaf age. PERMANOVAs showed that relative leaf age was a significant predictor of fungal (*p* = 0.002) and bacterial (*p* = 0.002) community dissimilarity.

Leaf age influenced the community composition of both fungi (PERMANOVA*, F*_2,71_ = 2.57, *p* = 0.002, R^2^ = 0.064) (Figure 4A) and bacteria (PERMANOVA, *F*_2,78_ = 3.08, *p* = 0.002, R^2^ = 0.070) (Figure 4B). In addition to leaf age, the combination of sampling timepoint and transect influenced both bacterial (PERMANOVA*, F*_2,78_ = 1.64, *p* = 0.002, R^2^ = 0.038) and fungal (PERMANOVA*, F*_2,71_ = 2.01, *p* = 0.036, R^2^ = 0.050) community composition. In betadisper tests, differences in diversity were not associated with differences in compositional variation between groups (fungi, *p* = 0.80; bacteria, *p* = 0.23), indicating that positive PERMANOVA results were not the result of heterogenous dispersion. Although relative leaf age, timepoint and transect all contributed to significant variation in microbial community structure, the contribution of these variables to changes in overall microbial community structure was relatively low – for bacteria, our model explained 10.78% of variation in community structure, and for fungi, our model explained 11.43% of the variation in community structure. Host individual did not predict changes in bacterial or fungal community composition.

### Relative abundance of bacterial genera, but not fungal genera, varied by leaf age in the field

**FIGURE 5:**
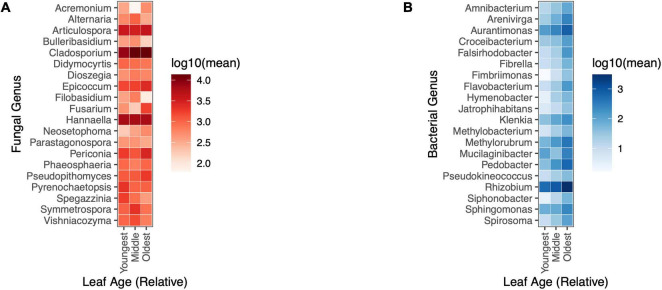
Changes in the relative abundance of bacterial genera, but not fungal genera, were evident by leaf age in the field. **(A)** Fungal taxa did not differ in abundance by relative leaf age in the field (univariate GLM (0/20), *p* > 0.05) **(B)** 14/20 of the most abundant bacterial taxa differed in relative abundance by leaf age in the field (univariate GLM (14/20), *p* < 0.05).

In the field, the relative abundances of bacterial taxa tended to vary by leaf age, yet no fungal taxa differed significantly in their relative abundance across leaves of different ages (univariate tests, *p*-adjusted < 0.05) (Figure 5A). 14 of the 20 most abundant bacterial genera differed in abundance between leaves of different relative ages (univariate tests, *p*-adjusted < 0.05). All significantly differentially abundant bacterial genera appeared to increase in relative abundance with increasing leaf age (Figure 5B).

### Leaf age influenced diversity, but not the relative abundance of fungi in the greenhouse

In addition to understanding how relative age influences fungal diversity in the field, we wanted to investigate temporal patterns of microbial diversity by leaf age, and in the absence of climate variation (which has been shown to structure microbial communities). Leaves from the greenhouse were not surveyed for bacterial diversity. Leaves from the greenhouse surveyed for fungal diversity by Illumina sequencing of ITS returned 5,578,116 reads and of those reads, 3,829,087 passed filtering. Each sample had an average of 38,677 reads per sample and DADA2 identified 3,244 unique ASVs (post-rarefaction), and all ITS reads aligned to the kingdom fungi.

**FIGURE 6:**
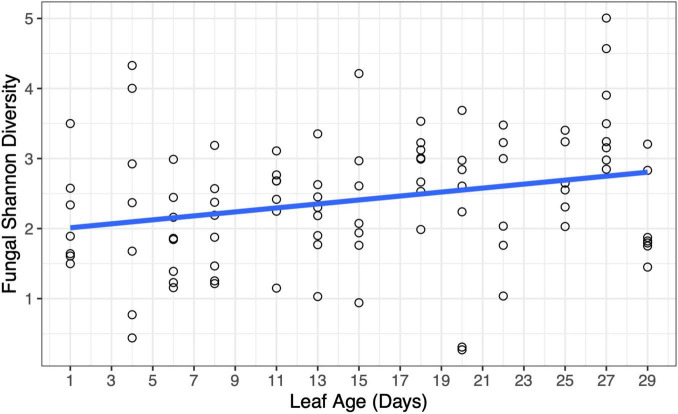
Changes in fungal diversity were evident at short time scales in the greenhouse. Fungal Shannon diversity increased with increasing leaf age (*p* = 0.0498). A model-fitted slope line was added to smooth points and visualize trends in fungal diversity by leaf age.

**FIGURE 7:**
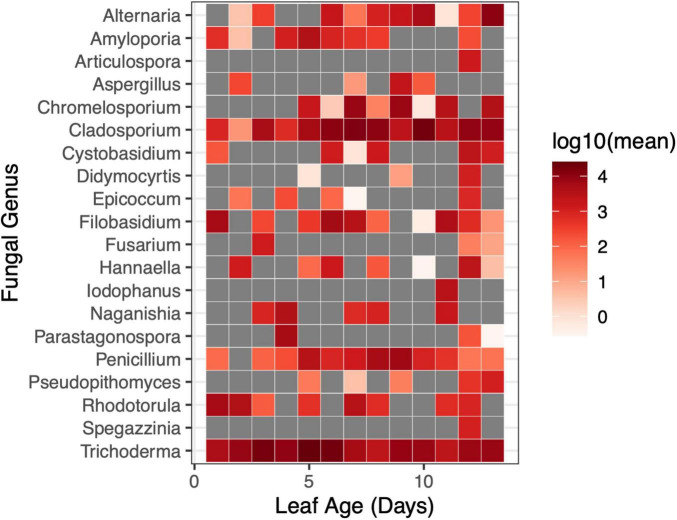
Changes in the relative abundance of fungal genera were not evident at short time scales in the greenhouse. The relative abundance of fungal genera did not differ by leaf age in the greenhouse (univariate GLM (0/20), *p* > 0.05). Gray squares indicated that a taxon was not present at the given sampling timepoint.

There was a weak relationship between leaf age and fungal Shannon diversity whereby fungal diversity increased with increasing leaf age (LMM, *F*_1,90.8_ = 3.95, *p* = 0.0498) (Figure 6); however, there was no support for a relationship with leaf age and fungal richness (LMM, *F*_1,91_ = 0.044, *p* = 0.83) (Supplementary Figure 3). Although fungal alpha diversity increased with leaf age, between-sample fungal diversity (beta diversity) did not differ by leaf age in the greenhouse, and leaf age explained little variation in fungal community structure (PERMANOVA, *F*_1,91_ = 1.40, *p* = 0.184, R^2^ = 0.016). None of the 20 most abundant fungal taxa significantly differed in abundance as leaves aged. Univariate tests could not detect statistical differences in fungal genera as a function of leaf age (univariate tests with Holm step down procedure, *p* > 0.05) (Figure 7).

## Discussion

Previous studies have shown that the age of a host influences the diversity of both bacteria and fungi – this trend has been evident across systems, including in plant systems (Lerner et al., 2006; Xu et al., 2009; Wagner et al., 2016; Biget et al., 2021; Doherty et al., 2021). Likewise, in the greenhouse study, we observed changes in fungal diversity as the host aged, with older leaves having higher fungal diversity. Over the course of 29 days, we were able to see a slight increase in fungal Shannon diversity (but not fungal richness) – there were more visible increases in diversity once leaves were 2 weeks old. Although previous studies have shown that changes in individual microbial taxa can occur at short time scales (Maignien et al., 2014), our study detected community-level changes in the leaf microbiome at time scales that have not previously been considered.

The effect of age on fungal diversity was modulated by the size of the host. This suggests that variation in fungal diversity within a given age class (e.g., younger leaves) can be further explained by differences in leaf size. Ecological theory suggests that the size of a habitat influences the diversity of the ecosystem (MacArthur and Wilson, 1967; Lawrence et al., 2018; Rybicki et al., 2020) – in our case, larger leaf size tended to yield more diverse and richer fungal communities but had no effect on the diversity or richness of bacterial communities. Positive relationships between the size of habitat (here, a leaf) and species diversity can result from extinction and colonization dynamics (Forney and Gilpin, 1989; Griffen and Drake, 2008) For example, it is possible that area is a more limiting factor for fungi colonization than bacteria, given their larger morphology, which could yield the trend we observed in our study. However, variation in host size is also related to changes in host age, physiology, and exposure to microbes, so this might not be an effect of area *per se* (MacArthur and Wilson, 1963, 1967). Given these results, future studies could consider sampling equal areas of the host to control for effects of area on microbial diversity. These results highlight the value of studies using leaf disks to control for the effects of leaf area on microbial diversity.

In addition to host age modulating the richness and diversity of microbes, we also saw that host age altered the community composition of both bacteria and fungi in the field, which has been observed throughout the literature (Lerner et al., 2006; Xu et al., 2009; Wagner et al., 2016; Biget et al., 2021; Doherty et al., 2021). In addition to seeing differences in community structure between leaves of different ages, we also saw considerably more variation in bacterial community structure in the youngest leaves, and less variation in older leaves – this suggests that there could be convergence in community structure as an individual leaf ages, or ecological drift early in the experiment. A similar trend, although less conspicuous, was observed in fungal communities, with younger leaves having more variation in community structure. Although leaf age was associated with differences in fungal community structure in the field, age accounted for relatively little variation in community structure. In the greenhouse, we saw no evidence for changes in fungal community composition by leaf age. In both cases, this suggests that there are many other factors that are likely shaping microbial communities, factors that we did not test in our study.

In the field, there were differences in the relative abundance of bacterial taxa in leaves of different ages, but there were no differences in the relative abundance of fungal taxa, either in the field, or in the greenhouse – however the paucity of observations of many fungal taxa across leaf ages may explain, in part, this non-significant finding in the greenhouse. Of the 20 most abundant bacterial taxa, 14 genera changed significantly by leaf age – in general, older leaves had higher abundances of bacterial taxa. More specifically, nitrogen fixing bacteria, like *Rhizobium spp.* and *Aurantimonas spp.* were present in higher abundances in older leaves. Because nitrogen fixing bacteria are often soil-borne, it would make sense that older leaves (which may have more contact with the soil, due to their length) would also have higher relative abundances of these taxa – however, in the field, plant tillers were matted in such a way that relative proximity to the soil was inconsistent with leaf age. *Methylobacterium spp.* was more abundant in older leaves. *Methylobacterium spp.* is a known contaminant of DNA extraction kits (Salter et al., 2014; Glassing et al., 2016) but is also known to grow endophytically within plant tissues (Andreote et al., 2006; Rossetto et al., 2011), and has been said to be a core part of plant microbiomes (Palberg et al., 2022). Within our two negative controls, we only detected 1 sequencing read which classified within the family Methylobacteriaceae – these results strongly suggest that *Methylobacterium spp.* reads from our plant leaf samples were not due to contamination from DNA extraction kits, and are, instead, present in or on plant leaves.

Our analyses of fungal and bacterial communities suggests that temporal changes in fungal communities may be more driven by changes in richness, whereas changes in bacterial communities may be more driven by changes in the relative abundance of bacterial genera. Leaf age had a greater effect on fungal richness than fungal Shannon diversity, and no fungal genera showed consistent or detectable changes in relative abundance between leaves of different ages in the field or the greenhouse. Conversely, bacterial richness did not differ by leaf age in the field, and there were significant differences in the relative abundance of bacterial taxa by leaf age. However, fungal taxa differed in Shannon diversity (which considers relative abundance) and community structure by leaf age, so the relative abundance of taxa still likely plays a role. Differences in species richness tell us nothing about what species are present, only that there are higher or lower numbers of unique species, whereas analysis of community composition identifies the unique taxa that make up a given community – both measurements are unique and important indicators of changes in community structure in a given ecological system. Results from these experiments corroborate previous studies showing changes in microbial (fungal and bacterial) diversity with host age (Lerner et al., 2006; Xu et al., 2009; İnceoğlu et al., 2011; Chaparro et al., 2013; Wagner et al., 2016; Biget et al., 2021; but see Doherty et al., 2021). Whether or not examining these changes at fine scales is relevant may vary by study system or microbial taxa considered.

Differences in the relative role that abundance and richness play in structuring fungal and bacterial communities may relate to microbe size and the spatial extent to which the microorganism can exert control on resources. For instance, fungi are larger than bacteria and can have spatially extensive morphologies — a single organism could exact influence at a larger spatial scale than bacteria. It is possible that space limitation in plant leaves creates competition between fungal species, which limits the number of taxa that can coexist (richness). Likewise, for smaller bacteria, space limitation may not be a significant factor, allowing taxa to coexist and increase in abundance as host ages; however, this hypothesis has not been empirically tested. In addition, inter-domain competition between fungi and bacteria is frequently observed (Faust and Raes, 2012; Vacher et al., 2016).

The role that leaf age plays in structuring microbial communities has been evident in foundational research looking at the plant leaf microbiome (Lerner et al., 2006; Xu et al., 2009; Wagner et al., 2016; Biget et al., 2021; Doherty et al., 2021). With increased capacity to study and characterize micro-communities with next generation sequencing, we are better able to understand how these communities change in response to abiotic and biotic variables. Our studies in the field and the greenhouse build upon previous work by showing how fungal and bacterial communities change in parallel across hosts of different ages in the field, while in the greenhouse examining these changes in absence of climate, and at very short time scales. Future studies could integrate microbial functional traits into studies of age-related changes in the leaf microbiome – these functional traits may reflect organism-environment interactions. For instance, a study examining the rice leaf microbiome found that there were higher abundances of siderophore-producing bacteria in nitrogen-limited soils (Thapa et al., 2017). Examining how fungal and bacterial functional traits change as a host ages may give us insight into the within-host (or external) factors shaping those microbial communities. Likewise, characterization of the microbiome and how it responds to endogenous or exogenous variables provides a foundation for targeted work examining interactions between specific taxa, which cannot be done in a direct way through community-level microbiome surveys.

## Data availability statement

The datasets presented in this study can be found in online repositories. The names of the repository/repositories and accession number(s) can be found below: https://www.ncbi.nlm.nih.gov/, PRJNA1030418, PRJNA1032011.

## Author contributions

JG: Conceptualization, Data curation, Formal analysis, Investigation, Methodology, Writing – original draft, Writing – review and editing. RG: Formal analysis, Writing – review and editing. JW: Formal analysis, Writing – review and editing, Funding acquisition. CM: Conceptualization, Funding acquisition, Methodology, Supervision, Writing – review and editing.
