# Supplementary material for: Leaf age structures phyllosphere microbial communities in the field and greenhouse
Source: Front Microbiol. 2024 Aug 14;15:1429166. doi: 10.3389/fmicb.2024.1429166 (PMC11349622; doi:10.3389/fmicb.2024.1429166)
Supplement: Supplementary file 1 [file Data_Sheet_1.pdf]

Supplementary Material

Leaf age structures phyllosphere microbial communities in the field and greenhouse

Julie K. Geyer*, Rita L. Grunberg, Jeremy Wang, Charles E. Mitchell

*** Correspondence:** Julie K. Geyer: jgeyer@email.unc.edu

1 Supplementary Figures and Tables

1.1 Supplementary Figures

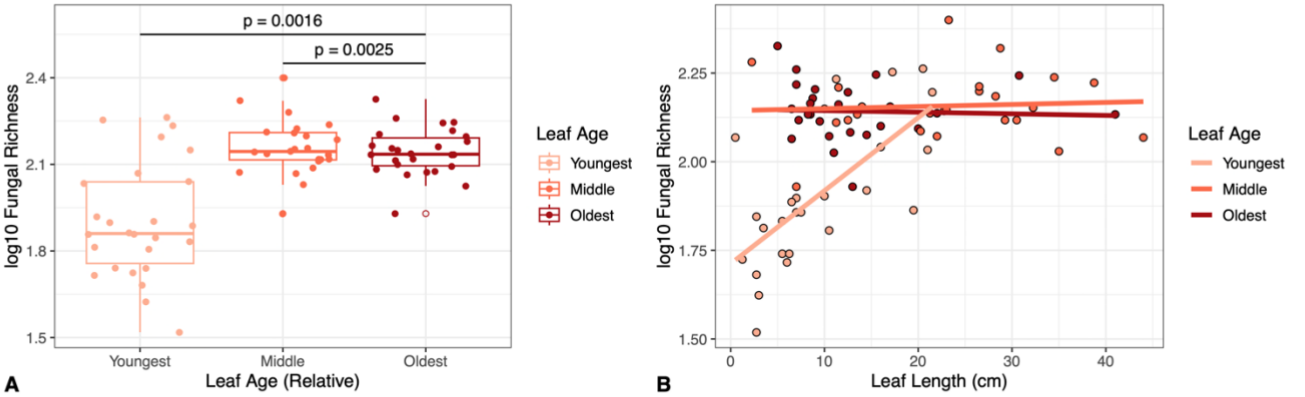

Supplementary Figure 1. The influence of leaf age on fungal richness depended on leaf length in the field. Panels show (A) the effect of relative leaf age on fungal richness ($p < 0.001$) and (B) the effect of leaf length and leaf age on fungal richness ($p < 0.001$).

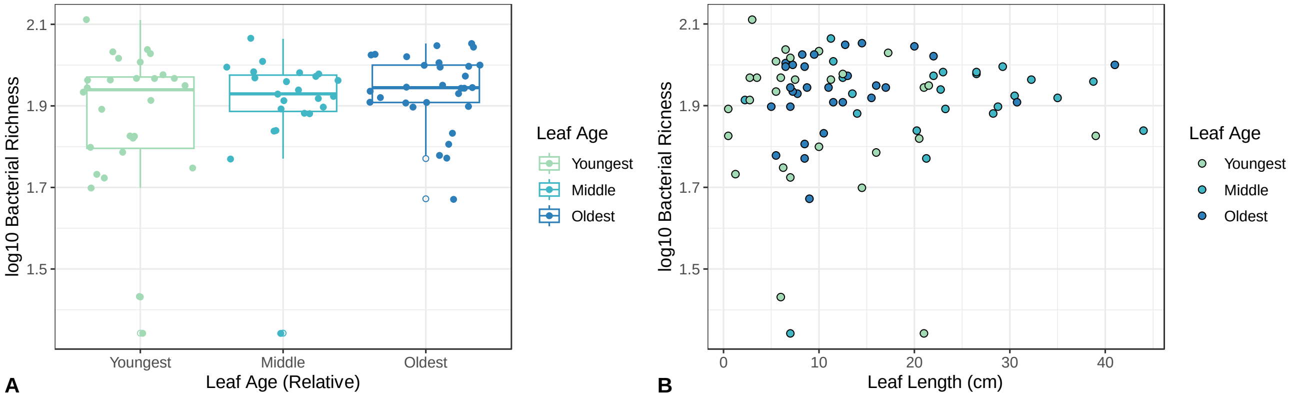

Supplementary Figure 2. Leaf age did not influence bacterial richness in the field. Panels show (A) the effect of relative leaf age on bacterial richness ($p = 0.23$) and (B) the effect of leaf length and leaf age on bacterial richness ($p = 0.69$).

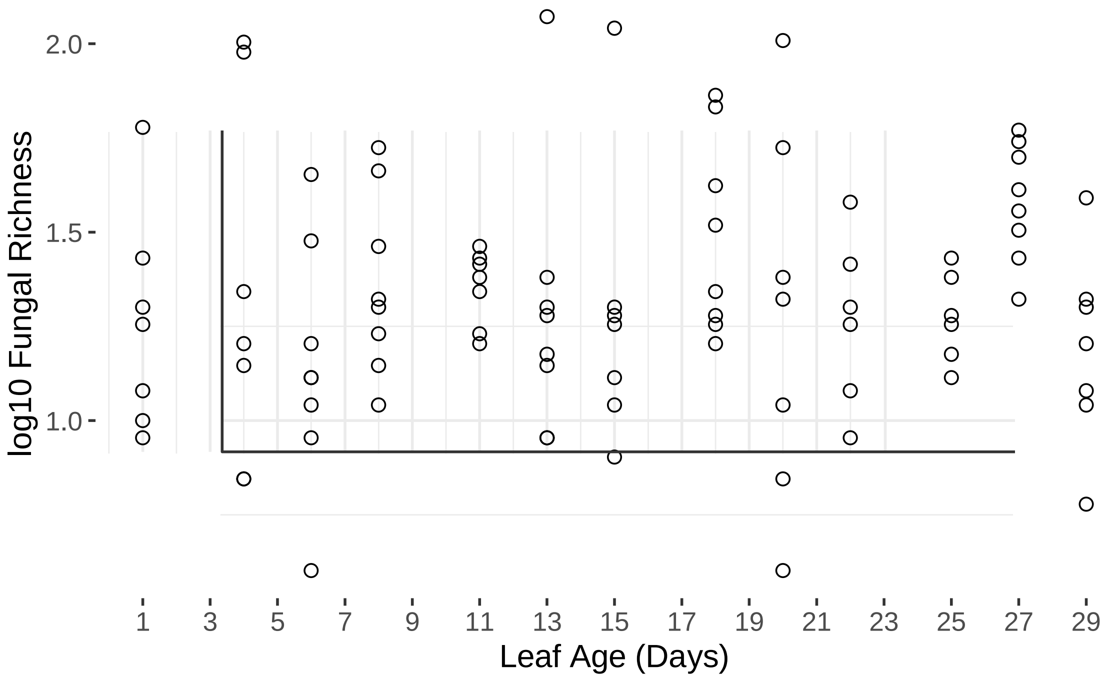

Supplementary Figure 3. Changes in fungal richness were not evident at short time scales in the greenhouse. A mixed effects model indicated that leaf age did not significantly influence fungal richness ($p = 0.83$).

1.2 Supplementary Tables

Fungal Genus	Total Number of Reads	Bacterial Genus	Total Number of Reads
Cladosporium	892,815	*Rhizobium*	129,025
Hannaella	500,328	*Aurantimonas*	33,844
Articulospora	282,497	*Pedobacter*	23,669
Periconia	153,546	*Methylorubrum*	17,281
Epicoccum	148,276	*Mucilaginibacter*	12,736
Pseudopithomyces	111,382	*Sphingomonas*	11,499
Pyrenochaetopsis	106,586	*Arenivirga*	10,456
Symmetrospora	99,915	*Klenkia*	9,124
Vishniacozyma	78,249	*Croceibacterium*	5,472
Spegazzinia	76,357	*Falsirhodobacter*	5,442
Didymocyrtis	66,134	*Flavobacterium*	5,351
Phaeosphaeria	65,555	*Spirosoma*	4,128
Fusarium	60,380	*Amnibacterium*	3,844
Alternaria	47,785	*Fibrella*	2,433
Dioszegia	44,324	*Methylobacterium*	2,308
Parastagonospora	30,625	*Pseudokineococcus*	2,152
Filobasidium	27,365	*Hymenobacter*	2,150
Neosetophoma	27,101	*Siphonobacter*	2,136
Bulleribasidium	25,816	*Jatrophihabitans*	1,495
Acremonium	23,756	*Fimbriimonas*	1,280

Supplementary Table 1. The total number of reads across all field samples in the 20 most abundant fungal and bacterial genera in the field.

Fungal Genus	Total Number of Reads
Trichoderma	931,978
Cladosporium	649,097
Chromelosporium	167,202
Filobasidium	150,455
Alternaria	148,172
Penicillium	143,195
Rhodotorula	113,945
Naganishia	62,642
Amyloporia	58,109
Cystobasidium	52,089
Parastagonospora	47,697
Hannaella	42,859
Aspergillus	18,445
Iodophanus	18,145
Pseudopithomyces	12,859
Articulospora	11,359
Fusarium	11,117
Epicoccum	9,749
Didymocyrtis	9,654
Spegazzinia	8,924

Supplementary Table 2. The total number of reads across all greenhouse samples in the 20 most abundant fungal genera in the field.
